# Evaluation of automated hematology analyzer DYMIND DH76 compared to SYSMEX XN 1000 system

**DOI:** 10.5937/jomb0-28836

**Published:** 2021-09-03

**Authors:** Milena Velizarova, Teodora Yacheva, Mariana Genova, Dobrin Svinarov

**Affiliations:** 1 Medical University of Sofia, Faculty of Medicine, Department of Clinical Laboratory, Sofia, Bulgaria; 2 UMBAL Alexandrovska, Clinical Laboratory & Clinical Pharmacology, Sofia, Bulgaria

**Keywords:** hematology analyser DYMIND DH76, performance evaluation, hematološki analizator DIMIND DH76, procena performansi

## Abstract

**Background:**

DYMIND DH76 (DYMIND BIOTECH, China) is a new automated hematology system designed to provide CBC count, including a 5-part WBC differential count, and its analytical performance should be assessed before adoption for clinical use.

**Methods:**

The analyzer was evaluated according to the International Council for Standardization in Haematology guideline. The purposes of this study were to assess its analytical performance in comparison to SYSMEX XN 1000 hematology analyzer currently used in our laboratory, as well as to compare the automated and manual WBC differential.

**Results:**

Within-run precision in all concentration ranges was very good with coefficients of variation (CVs) between 0.02% and 2.5% except for platelets over 500×109/L (CV 9.5%). Within-batch imprecision showed CVs lower the declared deviation ranges. Accuracy (defined as trueness) was excellent for all CBC and white cell differential parameters, compared with the state of the art%. Linearity was confirmed with excellent regression coefficients (0.999-1.000), even in the lowest values, and carryover was ≤ 1%. Comparison between DYMIND DH76 and SYSMEX XN 1000 was also very good with correlation coefficients (R2) for WBC (1.000), RBC (0.999), hemoglobin (0.999) and PLT over 50×10^9^/L (0.994) and R2 was lower but still acceptable (0.910) for PLT<50×10^9^/L. R2 for neutrophils, lymphocytes, eosinophils, basophils, and monocytes were 0.974, 0.982, 0.957, 0.625, and 0.836, respectively, in the comparison between the manual and DYMIND DH76 automated differential WBC counts.

**Conclusions:**

With excellent analytical performance and acceptable comparative analysis, DYMIND DH76 hematology analyser covered the predefined international standards and requirements and is fully appropriate for clinical application.

## Abstract (sr)

**Uvod:**

DYMIND DH76 (DYMIND BIOTECH, Kina) je novi automatski hematološki sistem napravljen da pokaže broj CBC, uključujući petodelni diferencijalni broj WBC. Pre ulaska u kliničku upotrebu, potrebno je da se ispitaju njegove analitičke performanse.

**Metode:**

U ocenjivanju analizatora korišćene su smernice Međunarodnog saveta za standardizaciju u hematologiji. Svrha ove studije je bila procena njegovih analitičkih performansi u odnosu na hematološki analizator SYSMEX XN 1000 koji se trenutno koristi u našoj laboratoriji, kao i da se uporedi automatski i ručni WBC diferencijal.

**Rezultati:**

Preciznost u toku ispitivanja u svim rasponima koncentracija je bila vrlo dobra sa koeficijentima varijacije (CV) između 0,02% i 2,5%, osim za trombocite preko 500×109/L (CV 9,5%). Nepreciznost unutar serije je pokazala da CV smanjuje deklarisani opseg odstupanja. Tačnost (definisana kao istinitost) je bila odlična za sve CBC i diferencijalne parametre belih ćelija, u poređenju sa procentom vrhunske tačnosti. Linearnost je potvrđena odličnim koeficijentima regresije (0,999-1,000), čak i u najnižim vrednostima, a prenos je bio ≤ 1%. Poređenje između DYMIND DH76 i SYSMEX XN 1000 je takođe bilo vrlo dobro sa koeficijentima korelacije (R2) za WBC (1,000), RBC (0,999), hemoglobin (0,999) i PLT preko 50×109/L (0,994), i R2 je bio niži, ali i dalje prihvatljiv (0,910) za PLT <50x109/L. R2 za neutrofile, limfocite, eozinofile, bazofile i monocite je bio redom 0,974; 0,982; 0,957; 0,625 i 0,836 u poređenju između ručnog i DYMIND DH76 automatskog diferencijalnog broja WBC.

**Zaključak:**

Sa odličnim analitičkim performansama i prihvatljivom uporednom analizom, hematološki analizator DYMIND DH76 je pokrivao unapred definisane međunarodne standarde i zahteve i u potpunosti je pogodan za kliničku primenu.

## Introduction

Clinical laboratories need to vigorously assess the performance characteristics and reliability of each analytical instrument before adoption for clinical use. DYMIND DH76 (DYMIND BIOTECH, China) is a new automated hematology analyser designed to report 29 parameters, including a 5-part WBC differential count, with a capacity for the analysis of 80 samples (CBC/DIFF) per hour. At the same time, it provides an enumeration of abnormal lymphocytes – ALY (%, #), large immature cells – LIC (%, #), platelet large cell ratio (P-LCR), and platelet large cell count (P-LCC). The analyzer works with anticoagulated (K_2_EDTA or K_3_EDTA) whole blood (venous and capillary), aspiration volume is 20 µL (mode CBC/DIFF), and pre-diluted samples could be processed in a special operation procedure. There is a possibility to interrupt the running series for urgent analyses (STAT function). DYMIND DH76 uses the impedance method with hydrodynamic focusing for counting WBC/BAS, RBC, and PLT, the non-cyanide colorimetric method for hemoglobin measurement, and semiconductor laser-based flow cytometry with light scattering as the principle for differential and total leukocyte count. Simultaneously, the analyzer provides information on the distribution of cells in three histograms, two 2D- and one 3D-scattergrams. Analytical characteristics declared by the manufacturer are presented in Table 1, Table 2, Table 3. DYMIND DH76 is able to generate flags in the presence of morphologically abnormal WBC, RBC, and PLT. The aim of this study was to assess the analytical performance of DYMIND DH76 with respect to: 1) manufacturer declared specifications (precision, reproducibility, carryover, and linearity); 2) comparison of DYMIND DH76 with the hematology analyzer SYSMEX XN 1000 currently used in our laboratory; 3) comparison between WBC differential count results obtained by DYMIND DH76 and microscopic morphologic (»manual«) examination. SYSMEX XN 1000 system (Sysmex, Kobe, Japan) determines erythrocyte and platelet counts by electrical impedance method and is able to perform an additional optical platelet measurement in the reticulocyte channel. For the WBC and differential counts, SYSMEX XN 1000 counter uses the flow cytometry method with fluorescent detection. WBC count is reported from the »WBC/BASO« channel, and control WBC data are generated from the independent »DIFF« channel. By comparing results produced by DYMIND DH76 with results obtained from an automated system based on different analytical principles and with manual differential WBC count, the performance of the new instrument would be reliably proven.

**Table 1:** Within-run imprecision results WBC, white blood cell count; RBC, red blood cell count; HGB, hemoglobin; HCT, hematocrit; MCH, mean corpuscular hemoglobin; MCV, mean corpuscular volume; RDW, red cell distribution width; PLT, platelets; NEU, neutrophils; LYM, lymphocytes; MON, monocytes; EOS, eosinophils; BAS, basophils; CV, coefficient of variation; SD, standard deviation; *Limits of acceptable within-run imprecision, based on current literature [1] [2] [3] [4]; **CV% declared imprecision by the DYMIND DH76 system manufacturer

Parameter	x̄	SD	CV%	CV% Limits of acceptable imprecision*	CV% limits of declared imprecision**
WBC normal level (3.5–10.5×10^9^/L)	4.8	0.07	0.05	2.5	2.0
WBC low level (<3.5×10^9^/L)	2.9	0.04	0.04	6.0	5.0
WBC high level (>11×10^9^/L)	25.3	0.25	0.20	1.5	5.0
NEU (%)	59.6	0.51	0.39	-	4.0
LYM (%)	31.0	0.69	0.57	-	3.0
MON (%)	6.3	0.46	0.36	-	2.0
EOS (%)	2.8	0.22	0.18	-	1.5
BAS (%)	0.15	0.07	0.06	-	0.8
NEU (×10^9^/L)	4.2	0.12	2.4	2.5	-
LYM (×10^9^/L)	2.1	0.07	3.4	3.5	-
MON (×10^9^/L)	0.6	0.05	7.8	8.5	-
EOS (×10^9^/L)	0.2	0.07	2.8	10	-
BAS (×10^9^/L)	4.8	0.13	2.9	20	-
RBC normal level (3.50–6.00×10^12^/L)	4.67	0.06	0.05	1.1	1.5
RBC low level (<3.5×10^12^/L)	1.61	0.04	0.03		-
RBC high level (>6.00×10^12^/L)	9.76	0.09	0.07		-
HGB normal level (120–180 g/L)	141.4	0.69	0.60	0.9	1.5
HGB low level (<100 g/L)	43.3	0.48	0.42	-	-
HGB high level (>180 g/L)	186.9	1.09	0.92	-	-
HCT (L/L)	0.43	0.01	0.01	1.2	-
MCV (fl)	93.0	0.58	0.33	0.6	1.0
MCH (pg)	30.2	0.38	0.25	1.1	-
RDW-CV (%)	13.3	0.07	0.05	2.0	-
PLT normal level (130–400×10^9^/L)	233.1	3.66	2.5	3.0	4.0
PLT low level (<30×10^9^/L)	2.7	1.05	0.9	4.5	8.0
PLT high level (>500×10^9^/L)	570.5	16.62	9.4	3.0	8.0

**Table 2:** Between-batch imprecision results (quality control material in the normal range). WBC, white blood cell count; RBC, red blood cell count; HGB, hemoglobin; HCT, hematocrit; MCH, mean corpuscular hemoglobin; MCV, mean corpuscular volume; RDW, red cell distribution width; PLT, platelets; NEU, neutrophils; LYM, lymphocytes; MON, monocytes; EOS, eosinophils; BAS, basophils; MPV, mean platelet volume; CV, coefficient of variation; SD, standard deviation. *Limits of acceptable within-run imprecision, based on current literature [1] [2] [3] [4]. **CV% declared imprecision by the DYMIND DH76 system manufacturer.

Parameter	x̄	SD	CV%	CV% Limits of acceptable imprecision*	CV% limits of declared imprecision**
WBC (×10^9^/L)	7.17	0.18	2.5	1.5	2
NEU (%)	58.8	1.32	2.2	2.5	-
LYM (%)	29.4	1.03	3.5	3.5	-
MON (%)	9.06	0.6	6.6	8.5	-
EOS (%)	2.72	1.03	1.9	10	-
BAS (%)	67.0	1.32	2.2	20	-
NEU (×10^9^/L)	4.2	0.12	2.8	2.5	-
LYM (×10^9^/L)	2.1	0.08	3.7	3.5	-
MON (×10^9^/L)	0.6	0.05	7.8	8.5	-
EOS (×10^9^/L)	0.19	0.07	1.5	10	-
BAS (×10^9^/L)	4.8	0.14	2.9	20	-
RBC (×10^12^/L)	4.99	0.06	1.3	1.1	1.5
HGB (g/L)	139.8	1.4	1.0	1.0	1.5
HCT (L/L)	0.45	0.005	1.0	1.4	-
MCV (fl)	90.8	1.09	1.2	0.8	-
MCH (pg)	27.9	0.46	1.7	1.5	-
RDW-CV (%)	16.9	0.17	1.0	2.0	-
PLT (×10^9^/L)	243.9	14.9	6.1	3.0	4
MPV (fl)	9.3	0.16	1.7	2.5	-

**Table 3:** Analytical accuracy (measured as trueness) on DYMIND DH 76 system, compared with state of the art for accuracy % State of the art for accuracy is based on the current literature [5] [6].

	n	x0	x̄	d%	State of the art (%)*
WBC (×10^9^/L)	30	6.88	7.17	-4.2	4.4
RBC (×10^12^/L)	30	4.86	4.99	-2.7	3.2
HGB (g/L)	30	138	139.8	-1.3	1.3
HCT (L/L)	30	0.43	0.45	-4.7	1.8
PLT (×10^9^/L)	30	247	243.9	1.3	6.4
MCV (fl)	30	90	90.8	-0.9	2.0
NEU (×10^9^/L)	30	4.3	4.2	2.3	3.2
LYM (×10^9^/L)	30	2.12	2.1	0.5	5.0
MON (×10^9^/L)	30	0.70	0.6	14.3	15
EOS (×10^9^/L)	30	0.17	0.2	-11.8	13
BAS (×10^9^/L)	30	4.65	4.8	-3.2	32*

## Materials and Methods

### Design of the study

In the present investigation, a total of 250 peripheral blood samples were analysed. For study purposes, fresh human whole blood samples, anticoagulated with K_2_EDTA, were used and processed no more than 2h after blood sampling. The blood samples were stored at room temperature to the time of analysis. Evidence of visible clogs or samples with insufficient volume was a reason to probe rejection. Samples were selected to cover normal conditions and a very broad range of different types of the underlying pathology and thus encompassed reference ranges, low and high results.

In this study, only DYMIND-specified reagents, controls, and calibrators were used, according to the manufacturer's instructions. Analytical characteristics of DYMIND DH76 and the comparison with SYSMEX XN 1000 were evaluated according to International Council for Standardization in Haematology (ICSH) guideline [7].

### Methods

Patient samples from the routine workflow, randomly selected in abnormally low, reference, and abnormally high analytical ranges were measured 10 consecutive times to assess reproducibility. For each hematologic parameter, the mean, SD, and CV% were calculated.

A single measurement repeated each day for a period of 30 days of the stabilized quality control material »CBC-5DMR Hematology Controls« – Normal (lotNo: BC1611), supplied by the manufacturer, was used to measure the total between-day precision for all included parameters. For each hematologic parameter mean value, SD and CV% were calculated.

Accuracy (assessed as trueness) was used to describe the closeness of a set of measurements to the true value [8] [9]. The use of a »true value« for the CBC is hard to apply in daily laboratory practice. For this reason, we studied the closeness of mean quality control material results, obtained by the analyses of quality control blood samples during the 30-day-period, to »target« values declared by the manufacturer of the quality control material. The percent deviation (d%) of the mean values from the »target« value was calculated for each parameter. Based on the current literature, our data were compared with state of the art for accuracy%.

Carryover was defined as the amount of analyte carried by the analyzer from one sample measurement into the subsequent measurement [7] [8] [6]. It was mainly of importance for carryover from high to low concentrations of Hb, RBC, WBC, and platelets. The carried biological material for HGB, RBC, WBC, and PLT was evaluated. The percentage carryover was assessed by analyzing a sample with a high concentration three times (H1, H2, H3) followed by analyzing a sample with a low concentration three times (L1, L2, L3). Percentage carryover is calculated as follows: %Carryover = L1-L3/H3-L3 100. For WBC, the low and the high values were 1.16×10^9^/L and 195.97×10^9^/L, for RBC – 2.68×10^12^/L and 7.25×10^12^/L, for HBG-86 g/L and 208 g/L, and for PLT-11×10^9^/L and 1787×10^9^/L, respectively.

The evaluation of linearity showed the ability of the hematology analyzer to provide a result that was proportional to the analyser measured over a defined concentration range [7] [8] [6]. For clinical purposes, the linear correlation between theoretical values and corresponded practical results, obtained by DYMIND DH76, was calculated especially for WBC, RBC, HGB, and PLT in low concentration ranges by the conduction of 1/2, 1/4, 1/8, 1/16 serial dilutions:

- The patient sample with the initial PLT value of 70×10^9^/L was diluted 1/2, 1/4, 1/8, 1/16 times to theoretical value of 4.3×10^9^/L.
- The patient sample with the initial WBC value of 3.9×10^9^/L was diluted 1/2, 1/4, 1/8, 1/16 times to a theoretical value of 0.24×109/L.
- The patient sample with the initial RBC value of 4.66×10^12^/L was diluted 1/2, 1/4, 1/8, 1/16 times to a theoretical value of 0.29×10^12^/L
- The patient sample with the initial HGB value of 120 g/L was diluted 1/2, 1/4, 1/8, 1/16 times to a theoretical value of 7.5 g/L.

Dilutions were performed manually with the equipment solvent DIL-A Diluent (DYMIND BIO-TECH, China). Dilutions were homogenized, then they were analyzed in duplicate. The test results were graphed and statistically analysed.

A comparison of the results from the evaluated DYMIND DH76 system with those obtained by the current hematology analyser SYSMEX XN 1000 was made for routine normal and abnormal samples in the laboratory.

Comparison between the manual and automated differential count of leukocytes was assessed. For the leukocyte differential count, the microscopic evaluation of a slide is currently regarded as the reference method [7] [1]. The manual differential count was observed by two independent qualified researchers in 400 cells per May-Grünwald-Giemsa stained slides at an optical microscope.

All results obtained during the study, including those of quality control materials, were classified, recorded, and stored in the analyzer software program and in computer program Excel worksheets (Windows office). Printed results for all samples analyzed on both hematology analyzers were labeled and collected.

### Statistical analysis

All statistical analyses were performed using IBM SPSS Statistics version 19.0 (IBM Corporation, Armonk, NY, USA). Patient sample correlations were calculated using Passing-Bablok regression and a difference comparison plot from the concordance study samples that were within the reportable range on both platforms. Pearson's correlation coefficient was employed to estimate linear relationships between the variables.

## Results

Results from within-run imprecision studies of blood samples on the DYMIND DH76 hematology system are shown in Table 1.

Results from between-batch precision studies, obtained by the analysis of quality control material in the normal range, are shown in Table 2.

Accuracy was measured as trueness and was used to evaluate the closeness of a mean of measurements to »target« values of quality control material. The percent deviation (d%) data were compared with state of the art for accuracy% Table 3.

**Figure 1:**
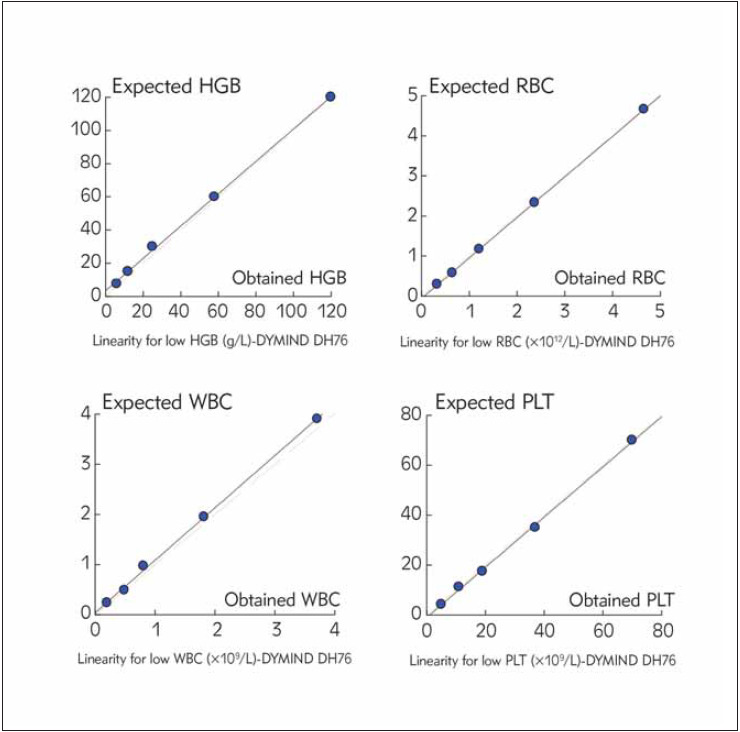
Linearity graph plots of HGB, RBC, WBC, and PLT

Linearity in low concentration ranges was verified for WBC, RBC, PLT, and HGB, and for all of them, it presented an excellent correlation coefficient (from 0.998 to 1.000) between expected theoretical and obtained values (Table 4, Figure 1 ).

Carryover was calculated according to the applied formula. For all of the analyzed parameters, carryover was less than 0.5% (Table 4).

**Table 4:** Carryover (%) and linearity (in low concentration range), DYMIND DH 76 WBC, white blood cell count; RBC, red blood cell count; HGB, hemoglobin; PLT, platelets.

	HGB (g/L)	RBC (×10^12^/L)	WBC (×10^9^/L)	PLT(×10^9^/L)
Carryover (%)	0%	0.4%	0.02%	0%
Declared carryover (%)	0.6%	0.5%	0.5%	1%
Linearity (r)	1.000	1.000	0.999	0.999

**Figure 2:**
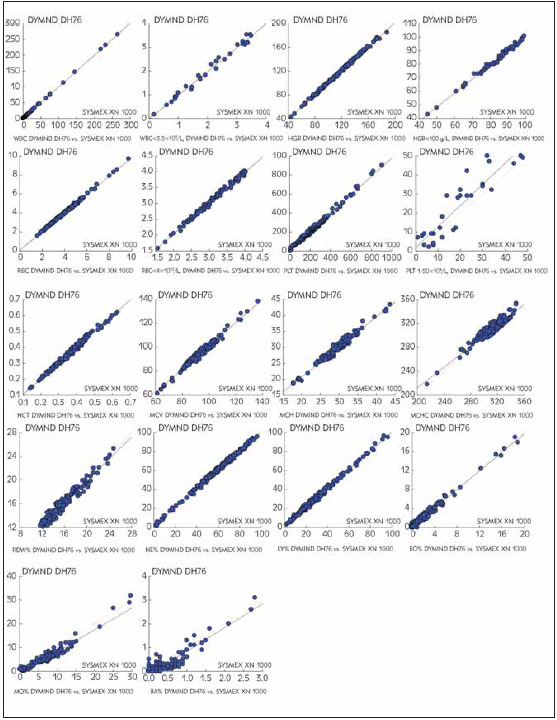
Correlation of complete blood count and white cell differential count results from the DYMIND DH 76 system compared with results from the SYSMEX XN 1000 system

Agreement between the obtained results from DYMIND DH76 and SYSMEX XN 1000 for complete cell count and differential WBC count was determined on 186 routine samples. Correlation statistics are presented in Table 5. On the regression scatter plots, DYMIND DH76 results were dependent (y) variables, and SYSMEX XN results were the independent (x) variables (Figure 2).

**Table 5:** Correlation results from the concordance studies WBC, white blood cell count; RBC, red blood cell count; HGB, hemoglobin; HCT, hematocrit; MCV, mean corpuscular volume; MCH, mean corpuscular hemoglobin; MCHC, mean corpuscular hemoglobin concentration; RDW, red cell distribution width; PLT, platelets.

Parameter	r	Slope	Y-intercept	SYSMEX XN1000 Mean	DYMIND DH76 Mean	Bias
WBC	1.000	1.02	-0.18	16.3	16.5	-0.2
WBC <3.5×10^9^/L, n=30	0.994	1.01	-0.04	2.14	2.11	0.03
RBC	0.999	0.97	0.09	4.05	4.02	0.03
RBC<3.5×10^12^/L, n=60	0.996	0.98	0.07	3.1	3.09	0.01
HGB	0.999	1.02	-2.10	116.9	117.3	-0.4
HGB<100g/L, n=60	0.996	1.04	-4.0	83.5	83.1	0.4
Hct	0.995	0.99	0.01	0.365	0.367	-0.002
MCV	0.987	0.99	2.20	92.4	94.1	-1.6
MCH	0.980	0.95	1.60	29.3	29.5	-0.2
MCHC	0.952	0.92	24.6	313.4	311.3	2.1
RDW-CV%	0.974	0.94	1.11	15.6	15.9	-0.3
PLT	0.994	0.97	3.1	234.7	230.7	4.0
PLT<50×10^9^/L, n=30	0.910	1.25	0.8	19.3	23.2	-3.9
% Neutrophils	0.998	0.99	1.17	56.9	57.5	-0.6
% Lymphocytes	0.997	0.99	1.26	30.2	31.2	-1.0
% Eosinophils	0.992	0.99	0.14	2.0	2.1	-0.1
% Basophils	0.855	1.00	-0.16	0.4	0.2	0.2
% Monocytes	0.965	0.95	-1.15	7.8	6.3	1.5

**Figure 3:**
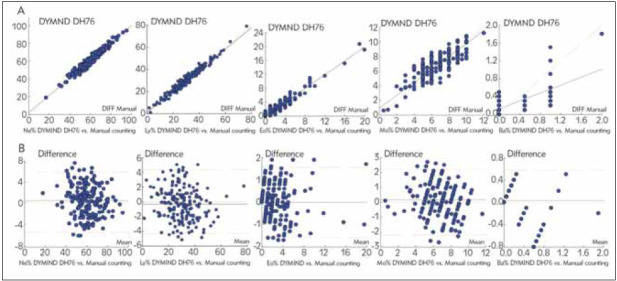
Correlation analysis (A) and difference plot estimation (B) of white blood cell differentials from the DYMIND DH 76 system compared with manual differentials

The correlation of the automated differential count of WBC with the manual method is shown in Table 6, and the difference plot estimation is shown in Figure 3.

**Table 6:** Correlation of white blood cell differential results from the DYMIND DH76 compared with manual white blood cell differential counts

Parameter	r	Slope	Y-intercept	Manual mean	DYMIND DH76 Mean	Bias
% Neutrophils	0.974	0.97	2.32	60.4	60.7	-0.3
% Lymphocytes	0.982	0.97	0.51	30.3	29.9	0.4
% Eosinophils	0.957	0.99	0.03	2.94	2.96	-0.02
% Basophils	0.625	0.44	0.13	0.20	0.17	0.03
% Monocytes	0.836	0.89	0.92	6.68	6.85	-0.17

## Discussion

In the current study, we evaluated the analytical performance characteristics (accuracy, precision, carryover, linearity in low concentration range) of the DYMIND DH 76 automated hematology system. At the same time, we performed a comparison between the studied DYMIND DH 76 and SYSMEX XN 1000 hematology analyzer, used routinely in our hospital laboratory.

The scope of examined hematological parameters with the purpose of evaluating precision depends largely on the type of laboratory which will use the hematology analyzer. For the needs of hospital laboratories, it is necessary to assess not only the reference area but also pathologically low and abnormally high areas of determination.

The determination of within-run imprecision was important to show the analyzer quality in test conduction and confirm the good repeatability of the analyzing samples with minimal appropriate differences. Within-run precision for the DYMIND DH76 system in all evaluated concentration ranges was very good and met the DYMIND DH76 specifications. Our results indicated low variation coefficients for withinrun imprecision in abnormally low and abnormally high white blood cell count, hemoglobin concentration, hematocrit, and platelet count samples. In contrast to our expectations, platelet count of 2.7×10^9^/L showed within-run imprecision of 0.9%, which was better than the previously documented studies and the declared deviation range (<10%) [6] [10] [11].

Between-batch precision was performed with a quality control material supplied by the manufacturer. We aimed to ensure the stability of the biological material during these 30 days of the study, and a quality control material was our appropriate choice. All coefficients of variation were excellent and were up to declared deviation ranges. The differential count of monocytes presented high CV% in the analyzed normal quality control level may be due to increased morphological variability of monocyte cells [12]. Maciel et al. [13] documented at the same manner the precision problem of monocyte count in hematology analyzers; however, the clinical significance of this imprecision is low.

The percent deviation data (d%) results, obtained from the accuracy study, were compared with state of the art for accuracy% from current literature [5] [6]. Accuracy (defined as trueness) of the DYMIND DH76 system was excellent for all CBC and white cell differential parameters (d% was comparable and even lower than the state of the art ones) [5] [6].

The carryover values in this analytical performance study were better compared with those recommended by the DYMIND DH76 manufacturer: the carryover for WBC, RBC, and HGB must be 0.5% and for PLT <1%. These data showed that there was not any transfer of material from one sample to the next sample, and the influence of contamination was close to zero. A lot of published studies recommend making a wash action between one sample and the next one to avoid a background count [12] [13] [14].

Linearity is an important measurement in the evaluation process. There should be a linear relation over a large concentration range at various dilutions for the parameter that is determined [7] [1] [14]. In the present study, our attention was narrowed to low thresholds of hematological parameters, where the clinicians have to make clinical decisions about the diagnosis and the appropriate treatment (whole blood transfusion, platelet transfusion) [15] [16] [17]. The results showed excellent linear relationships between theoretical and obtained values with regression coefficients r=0.999-1.000 for all of the analyzed parameters.

When comparing two hematology devices, the variability is usually greater than in intra-patient variation, mainly because analyzers are independent analytical systems, often with different methods of determination, as well as with calibrators and quality control materials from various manufacturers [7] [17] [2].

Our data indicated a very good positive correlation between CBC and white cell differential, obtained by the DYMIND DH 76 system and results obtained by the SYSMEX XN 1000 system. Most correlation coefficients for reported parameters were greater than 0.95. Difference plot estimation of blood count and white cell differential count results from the DYMIND DH 76 system compared with results from the SYSMEX XN 1000 system shows no significant discrepancy between the methods. The slightly lower correlation coefficient for platelet count in samples with PLT less than 50x10^9^/L (0.910) was found. According to the published studies, the accuracy of automated platelet counting depends on the applied automated method based on impedance, optical, fluorescent optical principles or flow-cytometry [10] [11] [15]. The SYSMEX XN 1000 system was able to use three of them, but for better comparison with DYMIND DH76, we performed the impedance method only.

As expected, cell types that occur in lower numbers in peripheral blood had a lower correlation coefficient compared to the other parameters included in the differential formula. The lower correlation coefficient for% basophils (0.855) was most likely due to the use of a more sensitive fluorescent method on the SYSMEX XN 1000 system. An even lower correlation coefficient for basophils was recently reported by Kaplan and co-workers for a different line of cell counters [14] [18].

Correlation of the white cell differentials obtained on the DYMIND DH 76 hematology analyzer with results of the reference method, manual 200cell counts by 2 researchers, was also very good. The performance was very good for neutrophils and lymphocytes (r>0.97), but it was clearly lower for monocytes (r=0.836) and for basophils (r=0.625). The decreased correlation coefficient for basophils is connected with the higher precision of the blood cell analyzers for cell types that occur in lower numbers in circulating blood because of the greater numbers of cells counted. In contrast, the decreased correlation coefficient for monocytes is not related to lower cell count in peripheral blood. In general, monocytes are difficult to identify due to their large morphological variations [8] [3] [4].

## Conclusion

Performance evaluation and comparative analyses of the automated DYMIND DH76 hematology system showed excellent analytical characteristics and laboratory results comparable to those obtained by the established laboratory practice analyzers. The DYMIND DH76 is useful for all laboratories, mainly in those with a large number of normal and abnormal samples. Its user-friendly workstation has complete patient data management, which is in compliance with good laboratory practices.

## Conflict of interest statement

All the authors declare that they have no conflict of interest in this work.
